# The mission to ensure continued funding for excellent basic research

**DOI:** 10.15252/embr.202357498

**Published:** 2023-05-25

**Authors:** Angus I. Lamond, Ivan Dikic, Andre Nussenzweig, Christoph W. Müller, Janet M. Thornton, Michael B. Yaffe

**Affiliations:** ^1^ Division of Molecular, Cell and Developmental Biology, School of Life Sciences University of Dundee Dundee UK; ^2^ Institute of Biochemistry II, School of Medicine Goethe University Frankfurt am Main Germany; ^3^ Center for Cancer Research National Cancer Institute Bethesda MD USA; ^4^ Structural and Computational Biology Unit European Molecular Biology Laboratory (EMBL) Heidelberg Germany; ^5^ European Molecular Biology Laboratory European Bioinformatics Institutes (EBI), The Wellcome Trust Genome Campus Cambridge UK; ^6^ MIT Center for Precision Cancer Medicine and Departments of Biological Engineering and Biology, Koch Institute Massachusetts Institute of Technology Cambridge MA USA

## Abstract

The surprising decision by Novo Nordisk Foundation (NNF) to discontinue funding for the Center for Protein Research in Copenhagen should prompt discussions about public and private commitment to support basic research.

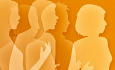

There is a growing trend for directing research funding in the life sciences toward “mission‐driven” projects. This can involve high‐profile initiatives, often generously supported by philanthropic donations from private foundations, companies, or wealthy individuals. Some of these initiatives have helped to establish new research centers and provided them with core funding for staff and equipment. These new centers are typically not set up as stand‐alone ventures. Rather, they are embedded within existing academic institutions, where they benefit from access to postgraduate students and the surrounding infrastructure and stimulating environment of the host campus.

Not surprisingly, the leaders of our universities, who are increasingly evaluated by their ability to attract research money as much as by their academic outputs, are enthusiastic to receive any and all such new sources of funding. Indeed, new initiatives are warmly welcomed when they bring additional financial resources that can support exciting new research opportunities, drive innovation, and open up new fields. Nonetheless, it should also be acknowledged that funding mission‐driven projects—together with the concomitant growing reliance on private financial support—can entail hidden risks and unwelcome consequences that we need to be aware of.

What are these hidden risks? For one, there could be decreased motivation to “think outside the box,” if the mission imposes a rather narrow focus, often influenced by current fashions and by the “top‐down” management nature of such projects. The corollary is that the mission increasingly emphasizes short‐term objectives and quickly generating tangible results, while discouraging long‐term investments that would allow researchers to tackle difficult and important problems where no immediate results are guaranteed. There are good examples in Europe of scientific breakthroughs being made in institutions where long‐term financial commitment allows outstanding scientists to tackle fundamental questions.

There is another risk that our political leaders view the trend toward private, philanthropic funding of research as a substitute for, rather than an addition to, funding basic science using taxpayers' money. We understand the pressure on governments to reduce costs while seeking the maximum value for money. However, it should be appreciated that most, if not all, research centers that receive core funding from private donors are in reality co‐funded and expect to also receive public funding. The reliance on private money therefore increases the risk that funding decisions are being made, “top down,” by independent and unaccountable bodies without an open peer‐review process and based on criteria that are neither transparent, fair nor consistent. Giving that the philanthropy partner has a *de facto* controlling stake in major decisions affecting university research centers, this raises important questions about ethics, accountability, and the management of potential conflicts of interest.

The issue at hand, in a nutshell, is how do we protect funding for excellent basic research, particularly when it does not involve a clear line of sight to a future commercial product or applied outcome? How do we ensure that fundamental science can continue to flourish if the evaluation processes do not regard excellence and/or outstanding performance as sufficient to justify continued support?

Some of these concerns came to the fore recently after the surprising decision by the Novo Nordisk Foundation (NNF), to discontinue providing core funding for the Center for Protein Research (CPR), which they had established in 2007 at the University of Copenhagen. The authors of this article were particularly surprised by this decision, because we have all served on the CPR's Scientific Advisory Board (SAB) and were familiar with their outstanding scientific achievements and pioneering contributions to science. At our most recent visit in 2022, the SAB was highly enthusiastic about the exciting future research plans put forward by CPR scientists, which we unanimously viewed as outstanding.

Novo Nordisk Foundation's decision to close the CPR was taken with no transparent, quinquennial review process, against the recommendation and report provided by the SAB, and with no opportunity for review, or appeal. Instead, it would seem that the NNF, which is the wealthiest foundation in the world that supports the life sciences (https://en.wikipedia.org/wiki/List_of_wealthiest_charitable_foundations), has decided to turn their back on funding the world‐class research at the CPR. They announced that they are considering new projects and a new mission instead, which is currently being decided within the foundation. In our opinion, this decision was unjustified on scientific grounds, given the outstanding achievements of the CPR scientists. Importantly, we are also concerned that the closure of the CPR will damage the future careers of the many talented young researchers who were recruited to work in Copenhagen with an expectation that funding would continue in return for excellent performance.

Building a world‐class center of excellence in any field of basic research is a hugely challenging task. It is not guaranteed by generous funding alone, but instead requires the dedication and exceptionally hard work by the scientific and administrative staff involved over many years. This makes the seemingly capricious decision to close the CPR all the more disappointing. Unfortunately, such a decision by an independent funding body to withdraw from long‐term core support for an outstanding research institute that has proven its value, is not unique. This happened previously, when, for example, Hoffmann‐La Roche shocked the immunology community by their decision in 2000 to close the famous and successful Basel Institute of Immunology.

We are not arguing here against the value of applied research, nor against funding from private organizations. We are also not opposed to targeted research projects with a clear, defined mission. Indeed, we are grateful that many philanthropies and other private organizations, including NNF, are willing to generously support science, including the establishment of new research centers and research missions.

Rather, we are concerned with the critical importance of ensuring continued funding to support scientists who pursue excellent, basic research, even without an overarching “mission,” or applied goal, beyond improving our fundamental understanding of biology. Put simply, our basic understanding of living systems is still sufficiently incomplete that we do not know where the next real breakthroughs will come from and we cannot predict with confidence what new approaches and innovations will be required to deliver future advances and insights. It is worth remembering that the science of recombinant DNA, including the revolutionary CRISPR technology, came from studying viral infections of bacteria, while the rapid development of vaccines for COVID during the pandemic was enabled by previous fundamental studies of mRNA interactions with fat.

In an era of mission‐driven science, we call for Europe to focus on the bigger mission, that is, to ensure that it fosters—and funds—a vibrant, basic research community for the benefit of society and future generations. We therefore ask our funders and politicians, when making funding decisions, to place the major emphasis on scientific excellence, while ensuring transparency and consistency. Researchers need the freedom to tackle challenging problems that may require long‐term support, regardless of any obvious immediate commercial or medical application. Consequently, public and private funding should be committed to provide long‐term support for high‐quality, basic science that creates new ideas and opportunities for the success of future translational projects and new research missions.

To help achieve this goal, we propose that international funding organizations, such as EMBO and the ERC, use their reputation and influence to lead an initiative to establish “best practice” standards for funding bodies, both public and private. While we respect the rights of private foundations to choose who and what to support, we advocate that all funding bodies and host institutions agree to adopt formal review procedures, along with safeguards against unilateral decisions that would lead to sudden loss of support for vulnerable, early‐career researchers. Eligibility for research centers that wish to apply for ERC or EMBO funding, for example, could be made contingent on adopting such procedures. Science, and in particular basic research, would greatly benefit if fundamental decisions about long‐term investments into basic research were made based on formal review with clear and transparent criteria.

## Disclosure and competing interests statement

The authors are all former members of the Scientific Advisory Board of the Center for Protein Research at the University of Copenhagen, Denmark.

